# Supplementary material for: The R251K Substitution in Viral Protein PB2 Increases Viral Replication and Pathogenicity of Eurasian Avian-like H1N1 Swine Influenza Viruses
Source: Viruses. 2020 Jan 2;12(1):52. doi: 10.3390/v12010052 (PMC7019279; doi:10.3390/v12010052)
Supplement: Supplementary file 1 [file viruses-12-00052-s001.zip › viruses-666310-supplementary.pdf]

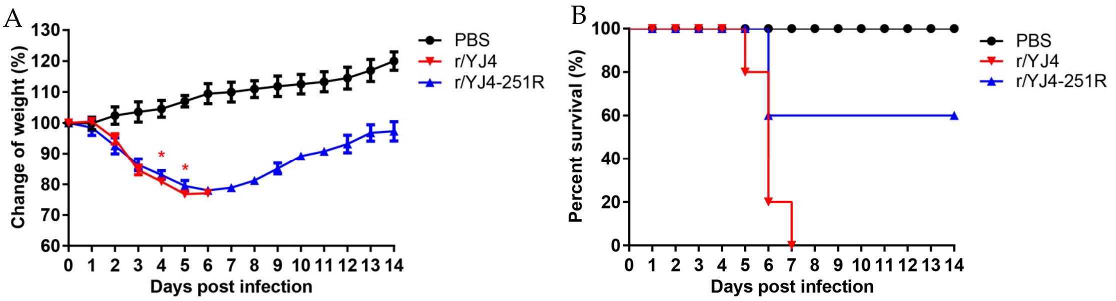

Figure S1. Weight changes and survival rates of mice infected with r/YJ4 and r/YJ4-251R viruses. Six-week-old female BALB/c mice were inoculated intranasally with r/YJ4 and r/YJ4-251R viruses at 10^6 $TCID_{50}$. Body weight change rate (**A**) and survival rate (**B**) in the infected mouse were continuously recorded for 14 days. Mice with weight loss of more than 25% of their initial body weight were euthanized humanely. Significant body weight changes of the mutant virus-inoculated mice compared with YJ4 virus-inoculated mice (*, $p < 0.05$).

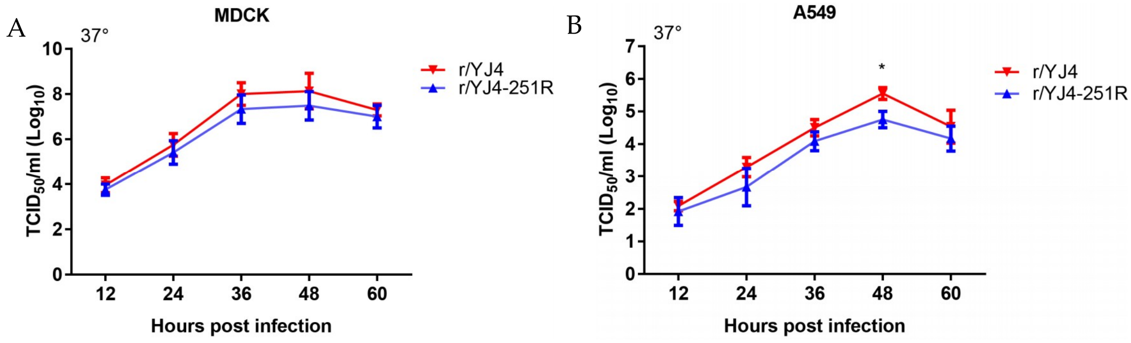

Figure S2. Growth Kinetics of r/YJ4 and r/YJ4-251R viruses in MDCK (**A**) and A549 (**B**) cells at 37 °C. MDCK cells (**A**) or A549 cells (**B**) were infected at an MOI of 0.01 with r/YJ4 and r/YJ4-251R viruses and cultured at 37 °C. Culture supernatants were harvested at 12, 24, 36, 48 and 60 hpi and subjected to $TCID_{50}$ assay in MDCK cells. Results are expressed as the means ± SD (n = 3) and the statistical significance was calculated using one-way ANOVA. *, $p < 0.05$.
